# Deciphering the Multifactorial Nature of *Acinetobacter baumannii* Pathogenicity

**DOI:** 10.1371/journal.pone.0022674

**Published:** 2011-08-01

**Authors:** Luísa C. S. Antunes, Francesco Imperi, Alessandra Carattoli, Paolo Visca

**Affiliations:** 1 Department of Biology, University Roma Tre, Rome, Italy; 2 Department of Infectious, Parasitic and Immune-Mediated Diseases, Istituto Superiore di Sanità, Rome, Italy; Monash University, Australia

## Abstract

**Background:**

*Acinetobacter baumannii* is an emerging bacterial pathogen that causes a broad array of infections, particularly in hospitalized patients. Many studies have focused on the epidemiology and antibiotic resistance of *A. baumannii*, but little is currently known with respect to its virulence potential.

**Methodology/Principal Findings:**

The aim of this work was to analyze a number of virulence-related traits of four *A. baumannii* strains of different origin and clinical impact for which complete genome sequences were available, in order to tentatively identify novel determinants of *A. baumannii* pathogenicity. Clinical strains showed comparable virulence in the *Galleria mellonella* model of infection, irrespective of their status as outbreak or sporadic strains, whereas a non-human isolate was avirulent. A combined approach of genomic and phenotypic analyses led to the identification of several virulence factors, including exoproducts with hemolytic, phospholipase, protease and iron-chelating activities, as well as a number of multifactorial phenotypes, such as biofilm formation, surface motility and stress resistance, which were differentially expressed and could play a role in *A. baumannii* pathogenicity.

**Conclusion/Significance:**

This work provides evidence of the multifactorial nature of *A. baumannii* virulence. While *A. baumannii* clinical isolates could represent a selected population of strains adapted to infect the human host, subpopulations of highly genotypically and phenotypically diverse *A. baumannii* strains may exist outside the hospital environment, whose relevance and distribution deserve further investigation.

## Introduction

*Acinetobacter baumannii* is an emerging human pathogen which causes a broad array of infections (e.g. pneumonia, urinary tract, bloodstream and skin infections) that account for about 10% of all nosocomial infections [1], [2], [3]. Since the 1980s, three main epidemic *A. baumannii* lineages, hereafter referred to as clonal complexes I, II and III (CC1, CC2 and CC3, respectively) have emerged and spread internationally throughout many geographical areas [4]. Both multi-locus sequence typing and comparative genomics analysis revealed that isolates belonging to the same CC are highly homogenous, suggestive of a recent clonal expansion [4], [5]. These three lineages are characterized by multidrug resistance (MDR), a phenotype that has been expanding alarmingly over the years, with frequent reports of *A. baumannii* strains resistant to almost all clinically relevant antibiotics [6], [7].

While the epidemiology and antibiotic resistance of *A. baumannii* strains has been extensively studied, limited information is so far available concerning the virulence and pathogenicity traits of this bacterium. Cohort and case-control clinical studies have shown that *A. baumannii* infections can be severe, and clinical observations have posed the question of the existence of a strain-dependent pathogenicity [8]. However, the molecular and genetic basis of *A. baumannii* virulence remains poorly understood, and only a few determinants have been demonstrated to be important for *A. baumannii* virulence *in vivo*. One of these is OmpA, an outer membrane protein which adheres to and is taken up by epithelial cells, where it can induce apoptosis [9]. OmpA is also implicated in resistance to complement and biofilm formation [10], [11]. Recently, other proteins have also been proposed to contribute to *A. baumannii* virulence. A phospholipase D has been demonstrated to be important for resistance to human serum, epithelial cell invasion and pathogenesis in a murine model of pneumonia [12], while a phospholipase C has been shown to enhance toxicity to epithelial cells [13]. Moreover, a transposon mutant in a gene for penicillin binding protein 7/8 showed reduced virulence in rat pneumonia and soft-tissue models, as well as reduced serum resistance [14].

Several other factors have been investigated as potential determinants of *A. baumannii* pathogenicity, but their role has not yet been definitively ascertained. Capsular polysaccharide has been implicated in serum resistance since mutants in its biosynthetic pathway fail to survive in human serum [15]. The extracellular polysaccharide poly-β-(1–6)-N-acetyl glucosamine (PNAG), synthesized by the *pgaABCD* locus, and the Csu pili play a role in biofilm formation [16], [17], while a homologue of the staphylococcal biofilm-associated protein (Bap) is required for the development of mature biofilm structures [18]. Quorum sensing has been also shown to contribute to biofilm formation [19], but did not affect virulence in the *Galleria mellonella* insect model of infection [20]. In addition, some but not all *A. baumannii* isolates show gelatinase activity and cause mannose-resistance hemagglutination [21]. Moreover, different *A. baumannii* isolates display diverse resistance to desiccation [22], a feature that can influence persistence in the hospital environment.

Finally, bacterial pathogenicity is intimately linked to the ability to use specific iron acquisition strategies, which are essential for pathogen survival and growth in the low-iron environment of the human host [23]. In addition to the well-characterized siderophore acinetobactin [24], genome investigations have recently shown that *A. baumannii* has the potential to express several iron-acquisition systems, including two other siderophores, one shared by all *A. baumannii* strains sequenced to date, with the exception of SDF, while the other is so far unique to ATCC 17978, two heme-uptake systems and a ferrous iron acquisition [25], [26].

The aim of the present work was to identify specific virulence determinant(s) that could contribute to the success of *A. baumannii* as a human pathogen, and to investigate whether the occurrence of such virulence determinants in representative strains of the two main clonal lineages, CC1 and CC2, could explain the increased ability of these lineages to cause infection and persist in the hospital setting. In this context, four *A. baumannii* strains of different origin (three clinical isolates and one isolate from a human body louse), belonging to diverse clonal complexes, whose genomes were completely sequenced and annotated, were comparatively analyzed by a combination of genome- and phenotype-based strategies. This led to the identification of a number of virulence factors that could contribute to the pathogenic potential of *A. baumannii*.

## Materials and Methods

### Ethics Statement

Serum was obtained from five healthy volunteers who gave their written informed consent to the study, which was approved by the Review Board of the Department of Biology of the University Roma Tre.

### Bacterial strains and culture conditions

Bacterial strains used in this study were *A. baumannii* AYE, ACICU, ATCC 17978 and SDF, and *Pseudomonas aeruginosa* PAO1 (ATCC 15692). AYE strain is an epidemic multidrug-resistant clinical isolate responsible for a nationwide outbreak in France in 2001 [27]; ACICU is an epidemic multidrug-resistant clinical isolate responsible for an outbreak in Rome (Italy) in 2005 [28]; ATCC 17978 was isolated in 1951 from a 4-month-old infant with fatal meningitis, and differs from AYE and ACICU in being susceptible to most common antibiotics [29]; SDF is a fully antibiotic-susceptible strain isolated from a human body louse [27]. Since *A. baumannii* is rarely found on the human skin, it has been proposed that the association of SDF with a non-human host is the result of the louse's ingestion of contaminated blood from an individual with undiagnosed *A. baumannii* bacteremia [30]. Strains AYE and ACICU are phylogenetically grouped inside CC1 and CC2, respectively, whereas ATCC 17978 and SDF represent two discrete and less common lineages within the *A. baumannii* population [4]. No isolates belonging to the CC3 have been completely sequenced to date, and thus representatives of this clonal lineage were not included in this study.

Bacteria were cultured in Luria-Bertani broth (LB), minimal broth M9 with succinate as carbon source [31], Chelex 100-treated trypticase soy broth dialysate (TSBD) [32] and casamino acid medium supplemented with 0.4 mM MgCl_2_ (CAA) [33]. When required, media were supplemented with either 50 µM 2,2′-dipyridyl or 50 µM FeCl_3_ to generate low- and high-iron growth conditions, respectively.

### *G. mellonella* killing assay

The *G. mellonella* virulence assay was performed as described previously [34], with minor modifications. *G. mellonella* caterpillars in the final instar larval stage (average weight 500±60 mg) were injected with 10 µl of serial ten-fold dilutions in saline solution of *A. baumannii* cells grown for 14 h at 37°C in TSBD. Bacterial colony counts on LB agar plates were used to estimate the number of viable cells in each inoculum. At least 20 larvae were inoculated per experiment, with a total of at least three dependent and three independent experiments per strain. Ten larvae per experiment were injected with 10 µl of sterile saline solution as a negative control. Larvae were incubated at 37°C in Petri dishes (five larvae per dish) and monitored for a three-day time period. Larvae were considered dead when they did not respond to gentle prodding [34]. When required, bacterial cells were heat-killed by incubation at 80°C for 60 min. Lethal dose 50% (LD_50_) values were calculated using GraphPad Prism and the following equation: Y = A+(1 - A)/[1+exp (B - G×lnX)], where X is the number of viable bacterial cells injected, Y the fraction of larvae killed by the bacterial solution, A is the fraction of larvae killed by the control solution, and B and G are curve-fitting constants automatically calculated by GraphPad Prism [34]. LD_50_ was calculated as the value of X that corresponds to Y = 0.5.

### Growth assays

Cells from overnight cultures in a given medium were normalized to an OD_600_ of 0.01 in the same medium and grown at 4, 25, 30, 37, 42, 45 or 50°C for 24 h with vigorous aeration (250 rpm). The culture cell density was determined every hour by measuring the OD_600_. Maximum specific growth rates (µ_max_) were determined by fitting growth data to a logistic growth curve using GraphPad Prism and the following equation: Y = Y_M_×Y_0_/((Y_M_−Y_0_)×e^−xk^+Y_0_), where Y_0_ and Y_M_ represent OD_600_ values at time points 0 and M, respectively, k is a constant calculated automatically by the program and x represents the time of growth (in hours). Each µ_max_ was calculated as the derivative of the equation for the time point of maximum growth. The coefficient of determination (R^2^) was used to verify the goodness of fit of the data to the equation.

### Resistance to iron starvation and production of iron-chelating compounds

Resistance to iron starvation was assessed on TSBD agar plates containing a linear gradient of the iron chelator 2,2′-dipyridyl, ranging from 0 to 500 µM, generated according to the agar gradient-plate technique [35]. *A. baumannii* strains were grown in TSBD at 37°C for 14 h and then inoculated with a sterile loop handle onto the plates, starting from the side with the highest concentration of the iron chelator. Plates were incubated at 37°C for 24 h.

Total iron-chelating activity and the amount of hydroxamate- and catechol-type groups were measured in filter-sterilized culture supernatants by the chrome azurol S (CAS) liquid assay [36], the Csàky assay as modified by Gillam [37] and the Arnow test [38], respectively.

### Hemolytic activity assays

Hemolytic activity was assessed using both agar plate and liquid assays. For plate assays, bacterial cells were grown in TSBD at 37°C for 14 h, normalized to an OD_600_ of 1 in sterile saline, and 5 µl aliquots were spotted on Columbia agar plates (Biokar Diagnostics) supplemented with 5% sheep or horse defibrinated blood (OXOID). Plates were incubated at 37°C for 48 h.

Hemolytic activity in culture supernatants was determined by incubating filter-sterilized supernatants from bacterial cultures grown in TSBD for 14 h in the presence of 10% (final concentration) sheep or horse defibrinated blood, previously washed several times with sterile ice-cold phosphate-buffered saline (PBS), pH 7.4. After 3 h incubation at 37°C with gentle agitation, intact erythrocytes were harvested by centrifugation at 1000 g and 4°C for 20 min. The amount of hemoglobin released in supernatants was evaluated by measuring the OD_545_. The percentage of hemolysis (P) was calculated using the equation P = (X−B)/(T−B)×100, and then normalized to the cell density (OD_600_) of the bacterial culture. X is the OD_545_ of the sample analyzed, while B and T represent the baseline and total hemolysis, i.e. the OD_545_ obtained with sterile TSBD and deionized water instead of culture supernatant, respectively [39].

### Phospholipase C activity assay

Extracellular phospholipase C activity was determined using the chromogenic substrate *p*-nitrophenylphosphorylcholine (PNPC) as described [40]. Briefly, 900 µl of a solution of 10 mM PNPC in 250 mM Tris-HCl, pH 7.2, 1 µM ZnCl_2_, 60% glycerol was added to 100 µl of filter-sterilized supernatants from cultures grown for 14 h in TSBD. The reaction mixture was incubated at 37°C for 24 h and the OD_405_ was measured.

### Proteolytic activity assay

Extracellular proteolytic activity was determined using the azoalbumin assay as previously described [41]. Briefly, 500 µl of a 1 mg/ml azoalbumin solution in Tris-HCl, pH 7.7, were added to 500 µl filter-sterilized supernatants from cultures grown in TSBD for 14 h, and then incubated at 37°C for 24 h. Trichloroacetic acid was added at 13% final concentration to precipitate the non-degraded protein. Samples were incubated at −20°C for 20 min, centrifuged at 15000 g for 10 min, and the OD_440_ of the resulting supernatants was measured.

### Biofilm assay

Biofilm formation was measured according to the microtiter plate assay [42]. Bacterial cells were grown in TSBD for 14 h and normalized to an OD_600_ of 1.0. Aliquots of 100 µl were transferred to a sterile 96-well polystyrene microtiter plate (12 wells per strain) and incubated at 37°C for 24 h. Planktonic cells were removed and the attached cells were gently washed three times with sterile PBS, air dried, and stained with 0.1% crystal violet solution for 15 min. After washing the wells four times with distilled water, the surface-associated dye was solubilized by adding 200 µl of 95% ethanol to each well. The dye solutions from three wells were pooled and the OD_540_ was measured.

### Surface motility assay

LB, CAA and TSBD plates containing 0.5% agarose were prepared and strains were stab-inoculated with a pipette tip to the bottom of the polystyrene Petri dish from bacterial colonies grown overnight in LB agar (1.5%) plates. The plates were closed tightly with parafilm to prevent drying and incubated at 37°C for 24 h. Swarming motility was observed at the air-agarose interface. Twitching motility was assessed by removing the agarose layer, staining the plates with a 0.1% crystal violet solution for 30 min, and measuring the diameter of the motility disk. A minimum of three independent experiments was performed.

### Assay for resistance to human serum

Resistance to human serum was measured according to the protocol of Kim *et al.* [11]. In brief, bacterial cells grown in TSBD for 14 h were washed and resuspended in PBS to an OD_600_ of 1.0. Human serum from five healthy individuals was pooled together and diluted in PBS to a 40% final dilution. Heat-inactivated serum was prepared by incubating the same serum at 56°C for 30 min. Bacterial suspensions were then added to human serum or heat-inactivated serum to obtain a bacterial cell concentration of ca. 1×10^7^ CFU/ml and samples were incubated at 37°C for 2 h. Viable counts were determined at 0 and 2 h time points, and three independent experiments were performed.

### Assay for resistance to desiccation

Resistance to desiccation was measured according to the protocol of Jawad *et al.* [22]. Bacterial cells were grown in TSBD for 14 h, washed twice with PBS and resuspended in distilled water to an OD_600_ of 1. Twenty µl of each suspension (ca. 2×10^7^ CFU) were deposited onto sterile 13 mm diameter rounded glass coverslips and placed in an uncovered petri dish in an airtight transparent plastic box (17 by 11 by 5.5 cm). The relative humidity inside the plastic boxes was maintained at 31% by the presence of a saturated salt solution of CaCl_2_ in an open 5 ml beaker [22]. For viable cell determination, at each sampling time point the glass coverslips were vortexed vigorously for 15 s in 2 ml of sterile distilled water, the cells were harvested by centrifugation (2,000×g for 10 min), resuspended in 200 µl of saline solution and 100-µl aliquots of these suspensions (or of appropriate dilutions) were plated onto LB agar plates by the spread plate method. Three glass coverslips were analyzed for each sample. In case of samples containing <10 CFU/coverslip, the absence of viable cells attached to the coverslip was confirmed by placing the coverslip in 2 ml of LB medium. Bacterial growth was never observed after 48 h at 37°C and 250 rpm.

### Statistical analysis

Statistical analysis was performed with the software GraphPad Instat, using One-Way Analysis of Variance (ANOVA) followed by Tukey-Kramer multiple comparison tests. Survival plots were generated by the Kaplan-Meier method and analyzed by the log-rank test. Differences having a *P* value <0.05 were considered to be statistically significant.

## Results

### Virulence in the *G. mellonella* model of infection

*G. mellonella* has recently been proposed as a simple and useful *in vivo* model to assess *A. baumannii* pathogenicity [20]. Accordingly, the *G. mellonella* model of infection was used to compare the virulence of *A. baumannii* strains AYE, ACICU, ATCC 17978 and SDF. A representative time-kill experiment is illustrated in Supporting Information (Fig. S1). The LD_50_ values were calculated for each strain at 24, 48 and 72 h post-infection (Table 1). For all strains, the LD_50_ values decreased with time of incubation, attaining the final value at 48 h. No killing was observed with heat-inactivated *A. baumannii* cells. No significant differences in virulence were observed between the epidemic strains AYE and ACICU and the non-epidemic strain ATCC 17978 (*P*>0.05), all three of which showed similar LD_50_ values (2.0−5.5×10^5^ CFU). These results are comparable to those obtained by Peleg *et al.* [20] for other clinical *A. baumannii* strains. In contrast, the non-clinical strain SDF showed insufficient larval killing to calculate the LD_50_, even at the highest inoculum size. Thus, the estimated LD_50_ for SDF represents a theoretical approximation to the actual LD_50_ value, and can confidently be considered to be at least 100-fold higher than the LD_50_ values for the three clinical strains.

**Table 1:** *G. mellonella* killing by *A. baumannii* strains.

Strain	LD_50_ (± SEM) a		
	24 h	48 h	72 h
AYE	7.51 (±0.68)×10^5^	2.01 (±0.05)×10^5^	1.98 (±0.04)×10^5^
ACICU	6.64 (±0.26)×10^5^	5.58 (±0.09)×10^5^	5.54 (±0.11)×10^5^
ATCC 17978	3.92 (±0.10)×10^5^	2.42 (±0.13)×10^5^	2.38 (±0.12)×10^5^
SDF b c	4.99 (±0.31)×10^7^	4.84 (±0.06)×10^7^	4.84 (±0.06)×10^7^

a The LD_50_ values are expressed in CFU and were calculated as described in Materials and Methods after 24, 48 and 72 h of infection with *A. baumannii* cells. Values are the mean (± SEM) of at least three independent experiments and were inferred from a regression curve calculated from at least six different infecting inocula for each experiments.

b Since SDF causes insufficient killing of larvae for experimental determination of the LD_50_ even at the highest infecting dose (i.e. 4×10^7^ CFU/larva), the reported LD_50_ is a theoretical estimate generated by GraphPad Prism.

c The difference between the LD_50_ values of SDF and those of clinical strains is statistically significant (*P*<0.001) at every time point.

### Growth kinetics

In order to establish the optimal conditions for *in vitro* growth of the different *A. baumannii* strains, the growth kinetics were determined for each strain at temperatures ranging between 4 and 50°C in different minimal and complex growth media, namely LB, M9 minimal medium, CAA and TSBD.

**Figure 1:**
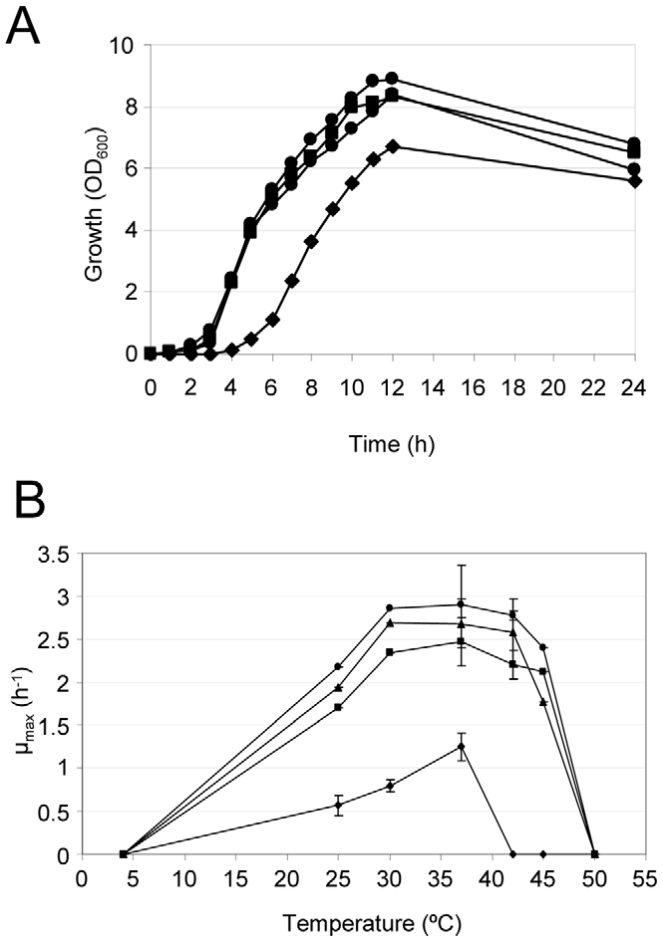
*A. baumannii* growth kinetics. (A) Growth (OD_600_) of *A. baumannii* strains at 37°C in TSBD medium. (B) Temperature-dependent growth kinetics, defined as maximum growth rates (µ_max_) of *A. baumannii* strains grown in TSBD medium at the different temperatures. Values represent the mean (± standard deviation, SD) of three independent experiments. Symbols: AYE, squares; ACICU, circles; ATCC 17978, triangles; SDF, diamonds.

SDF grew slower than the other strains in all the media tested, with TSBD being the only medium supporting the growth of SDF to levels almost comparable to those of the other strains (Fig. 1; data not shown). In general, TSBD and LB were found to be the preferential media for growth of *A. baumannii*, with maximum growth rates (µ_max_) of the clinical strains being slightly higher in TSBD (average of 2.8 h^−1^ at 37°C) than in LB (average of 2.5 h^−1^ at 37°C). When grown at 37°C in TSBD, strains entered stationary growth phase after ca. 10–12 h, with a doubling time of ca. 40 min for clinical strains and 100 min for SDF.

All *A. baumannii* strains of clinical origin grew at temperatures ranging between 25 and 45°C, with maximum growth rates at 37°C. In contrast, growth of the non-human SDF strain was impaired at temperatures higher than 37°C (Fig. 1), a feature that would hamper growth in humans during pyrexia.

### Iron-uptake capability

**Figure 2:**
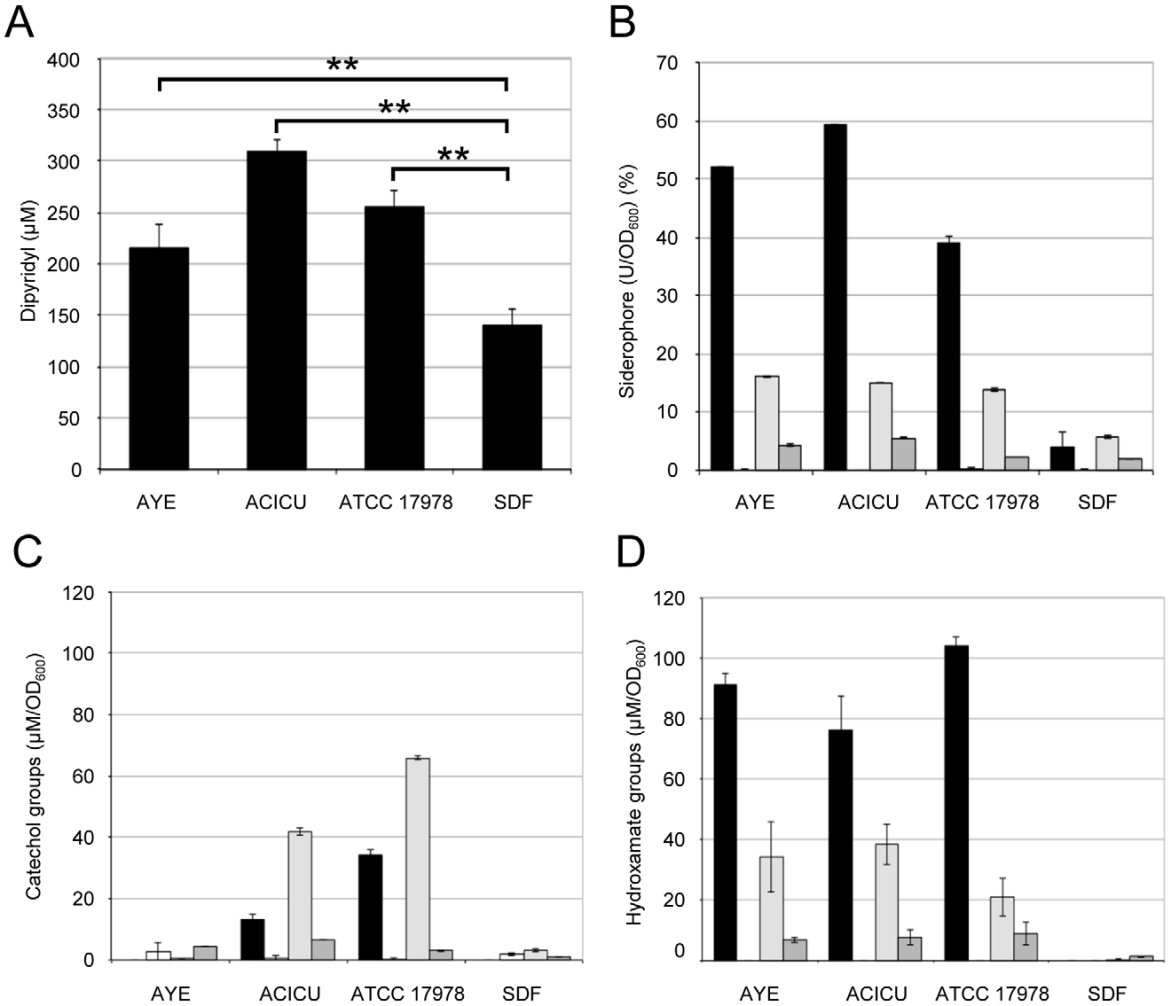
Iron-uptake capability. (A) Growth of *A. baumannii* strains on 2,2′-dipyridyl gradient (0–500 µM) TSBD agar plates. The ordinate shows the minimal inhibitory concentration of the iron chelator 2,2′-dipyridyl. (B) Iron-chelating activity in culture supernatants of *A. baumannii* strains grown in CAA supplemented with either 50 µM 2,2′-dipyridyl or 50 µM FeCl_3_ (black and white bars, respectively) and TSBD supplemented with 50 µM 2,2′-dipyridyl or 50 µM FeCl_3_ (light and dark gray bars, respectively). Values are expressed as percentage of siderophore units (U) normalized to the cell density (OD_600_) of the bacterial culture. Amount of catechol (C) and hydroxamate (D) groups in the supernatants of *A. baumannii* cultures. Values are expressed as concentration (µM) of hydroxamate or catechol groups normalized to the OD_600_ of the bacterial culture. Growth conditions and symbols correspond to those described in the legend to panel B. Values represent the mean (±SD) of two independent experiments, each including three biological replicates. ** *P*<0.01 (ANOVA).

The capability of the different *A. baumannii* strains to resist iron starvation was compared by growing the strains on TSBD agar plates in the presence of a gradient of the iron chelator 2,2′-dipyridyl (0–500 µM) (Fig. 2A). Considerable differences in resistance to iron starvation were found between strains, with clinical strains showing significantly higher capability to overcome iron starvation than the non-human isolate SDF (Fig. 2A; *P*<0.01).

In order to correlate the observed iron starvation resistance profiles to the production of iron-chelating compounds, the different *A. baumannii* strains were compared for total iron-chelating activity released in culture supernatants, as well as for production of iron-regulated molecules containing chemical groups involved in iron chelation, namely hydroxamates and catechols. To this aim, *A. baumannii* strains were grown in poor (CAA) or rich (TSBD) medium supplemented with either 50 µM 2,2′-dipyridyl or 50 µM FeCl_3,_ as iron-depleted and iron-replete growth conditions, respectively. While CAA is one of the reference media used for iron-uptake studies, TSBD was chosen since it supports growth of SDF to levels comparable to the other strains (Fig. 1).

With the exception of SDF, all strains showed iron-chelating activity in the supernatant of cultures grown under iron-depleted growth conditions (Fig. 2B), consistent with the genomic predictions. With regard to the chemical nature of the iron-chelating groups, all the siderophore-producing strains secreted comparable amounts of hydroxamate groups, while AYE showed only marginal catechol production with respect to ACICU and ATCC 17978 (Figs. 2C and D). Notably, the ratios between hydroxamate and catechol groups released by strains ACICU and ATCC 17978 were significantly different for the two growth media (*P*<0.001). While both strains produced higher amounts of hydroxamates than catechols in CAA, the opposite occurred in TSBD (Figs. 2C and D), indicating that the composition of the growth medium may affect the production of the different iron-chelating compounds.

### Hemolytic activity

Although *A. baumannii* is generally considered to be a non-hemolytic species [43], genomic analysis revealed the presence of two phospholipase C genes and a number of hemolysin-related genes in all the *A. baumannii* strains sequenced to date (Table S1 and ref. 19).

**Figure 3:**
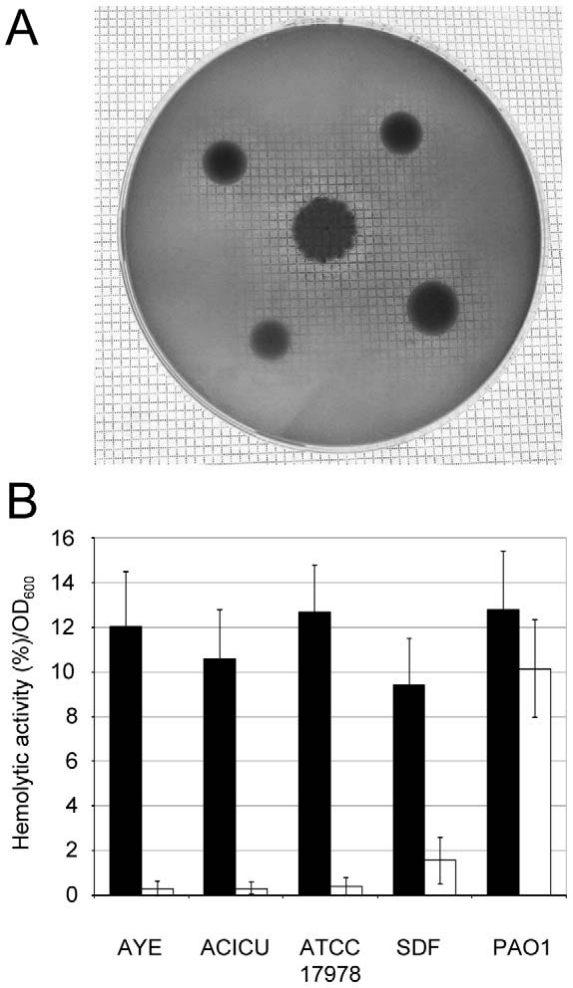
Hemolytic activity. (A) Hemolysis on 5% horse blood Columbia agar plates. *A. baumannii* strains are, from top left in clockwise order: AYE, ACICU, ATCC 17978 and SDF. *P. aeruginosa* PAO1 was spotted on the center of the plate as positive control for hemolysis. Hemolysis is evidenced by the transparent halo around the colony. (B) Hemolytic activity on horse and sheep erythrocytes (black and white bars, respectively) of cell-free supernatants from *A. baumannii* cultures grown in TSBD medium. Hemolytic activity was normalized to the OD_600_ of the bacterial culture. Values represent the mean (± SD) of two independent experiments, each including three biological replicates.

In order to assay the hemolytic activity, *A. baumannii* strains were grown on 5% sheep or horse blood agar plates. Intriguingly, all strains caused appreciable hemolysis of horse but not sheep erythrocytes (Fig. 3A; data not shown). To quantify extracellular hemolytic activity, filter-sterilized bacterial supernatants from TSBD cultures were incubated with horse and sheep defibrinated blood. Whereas no hemolysis was detected in the presence of sheep blood, all strains caused hemolysis of horse erythrocytes to levels comparable to that of *P. aeruginosa* PAO1, which was used as a positive control (Fig. 3B).

### Phospholipase C and exoprotease production

**Figure 4:**
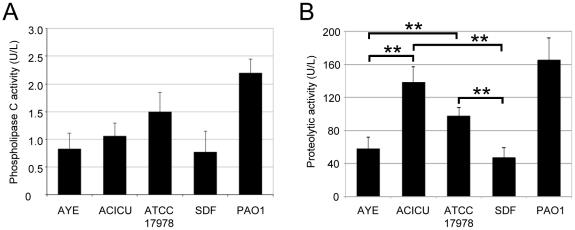
Production of virulence-related exoproteins. Phospholipase C (A) and total proteolytic (B) activities in filter-sterilized supernatants of the different *A. baumannii* strains grown for 14 h in TSBD at 37°C. Activities are expressed in U/L of culture supernatants and normalized to the OD_600_ of the bacterial cultures. *P. aeruginosa* PAO1 was used as positive control for phospholipase C and proteolytic activities. Values represent the mean (± SD) of three independent experiments. ** *P*<0.01 (ANOVA).

The genomes of the sequenced *A. baumannii* strains contain two genes putatively coding for a phospholipase C and several genes encoding putative secreted proteolytic enzymes (Table S1). Therefore, phospholipase C and exoprotease activities were compared in culture supernatants of the different *A. baumannii* strains. All strains showed phospholipase C and protease activities (Fig. 4). Whereas no statistically significant differences in phospholipase C levels were observed among isolates (*P*>0.05), ACICU and ATCC 17978 showed higher protease activities than AYE and SDF (*P*<0.01; Fig. 4B).

### Biofilm formation

**Figure 5:**
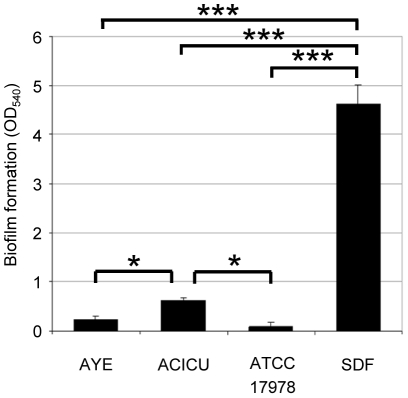
Biofilm formation. Biofilm formation on 96-well polystyrene microtiter plates by *A. baumannii* strains grown statically in LB medium for 24 h. Values represent the mean (± SD) of five independent experiments performed in quadruplicate. * *P*<0.05, *** *P*<0.001 (ANOVA).

Analysis of the *A. baumannii* genomes indicated that several genes predicted to encode factors responsible for promoting and regulating biofilm formation, e.g. pili and quorum sensing, were differently distributed among *A. baumannii* strains [5], [27], [28]. Biofilm formation was compared among the different *A. baumannii* strains by measuring cell adhesion to the surface of polystyrene microtiter plate wells after static incubation at 37°C for 24 h. Under these conditions, the non-clinical strain SDF showed ≥10-fold higher ability to adhere to the surface and form biofilms with respect to the clinical strains (*P*<0.001; Fig. 5). Among these, the ACICU strain, representative of CC2, exhibited significantly higher biofilm formation than the AYE strain, representative of CC1, and the non-epidemic strain ATCC 17978 (*P*<0.05; Fig. 5), in line with the recent observation that biofilm formation capability is higher in isolates belonging to CC2 compared with CC1 isolates [44].

### Surface motility

The *A. baumannii* genomes contain several genes and gene clusters putatively responsible for type IV pilus-mediated twitching motility. Compared with the epidemic strains AYE and ACICU, strain ATCC 17978 lacks three genes predicted to be involved in pilus adhesion, whilst SDF lacks most genes essential for pilus biogenesis and functioning (Table S2).

**Figure 6:**
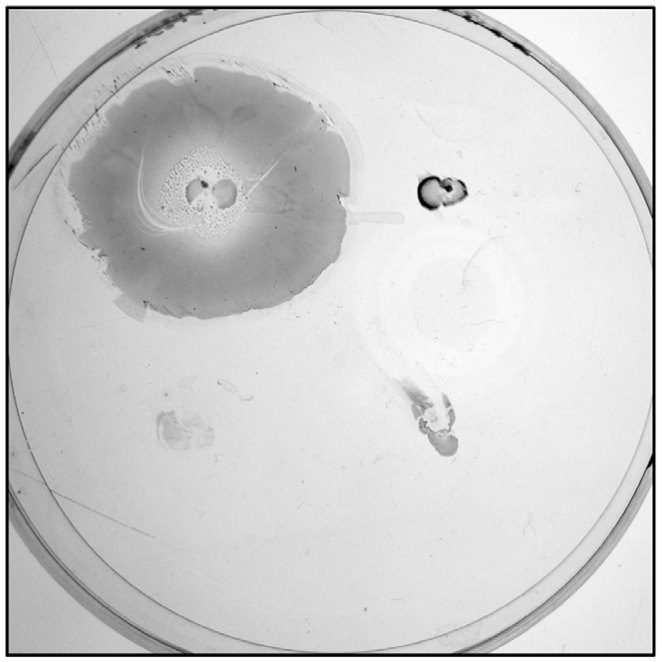
Twitching motility. *A. baumannii* twitching motility after 24 h of growth at 37°C in TSBD medium. *A. baumannii* strains are, from top-left in clockwise order: AYE, ACICU, SDF and ATCC 17978. The plate shown in the figure is representative of three independent experiments giving similar results.

Surface motility was assessed on LB, CAA and TSBD plates containing 0.5% agarose. After incubation at 37°C for 24 h, twitching motility was measured in terms of the bacterial spread at the agarose/petri plate interface [45]. Twitching was observed for AYE in LB and TSBD, but not in CAA plates, while the other strains did not twitch in any of the tested media (Fig. 6 and data not shown). Moreover, different forms of swarming were detected at the air/agarose interface for ATCC 17978 in CAA and for AYE in TSBD medium (Fig. S2).

### Resistance to human serum and desiccation

**Figure 7:**
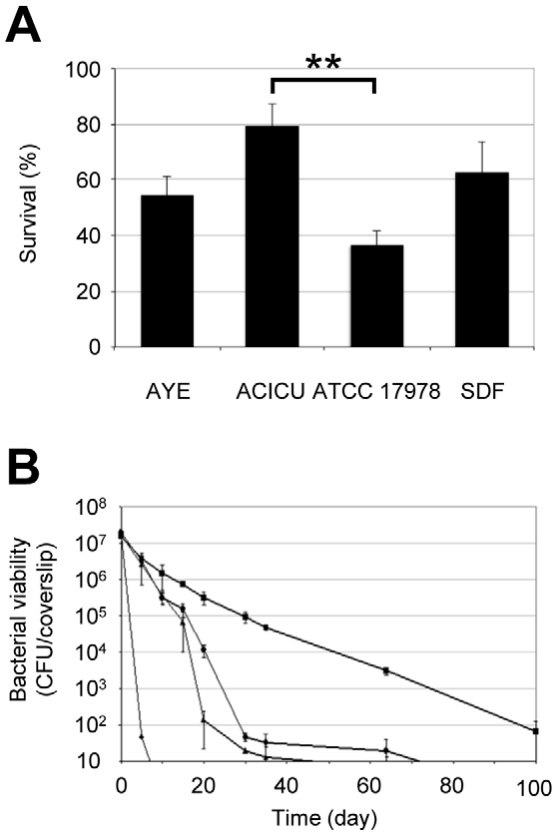
Resistance to human serum and desiccation. (A) Serum resistance of *A. baumannii* strains. Resistance was determined as the percentage of survival in 40% non-heated serum relative to survival in heated serum. Values represent the mean (± SD) of five independent experiments. (B) Resistance to desiccation of *A. baumannii* strains: AYE (squares), ACICU (circles), ATCC 17978 (triangles) and SDF (diamonds). Strains were inoculated onto 13-mm diameter rounded glass coverslips and incubated at 22°C and 31% relative humidity. A starting bacterial inoculum of 2×10^7^ CFU per coverslip was used. Values represent the mean (± SD) of three independent experiments. The lower detection limit of the assay is 10 CFU per coverslip. ** *P*<0.01 (ANOVA).

The different *A. baumannii* strains were compared for the ability to survive for long periods on dry surfaces and the ability to avoid the bactericidal activity of human serum. Overall, ACICU, AYE and SDF displayed a comparable resistance to human serum, while ATCC 17978 showed lower survival in the presence of human serum, a difference which was statistically significant only with respect to ACICU (*P*<0.01; Fig. 7A). On the other hand, the *A. baumannii* strains showed a considerably different ability to survive on dry surfaces (Fig. 7B). The louse strain SDF was strongly impaired in its ability to resist desiccation (survival time less than 10 days). The non-epidemic strain ATCC 17978 and the representative CC2 strain ACICU showed comparable trends of survival (survival time of 40–70 days). Notably, the representative CC1 strain AYE showed extremely high resistance to desiccation, surviving on dry surfaces for more than 100 days (Fig. 7B).

## Discussion

*A. baumannii* is an emerging human pathogen responsible for a broad array of nosocomial infections. Its ability to persist in the hospital setting and to steadfastly acquire antibiotic resistance has become a global concern for the medical community [1], [3]. Most studies have focused on the epidemiology and evolution of antibiotic resistance of *A. baumannii*; however, the basis of its virulence remains ill-defined.

Recently, Peleg and coworkers demonstrated the suitability of *G. mellonella* as a model to study *A. baumannii*-host interactions [20]. This simple animal model of infection was used in the present study to compare the pathogenicity of four *A. baumannii* strains with completely sequenced genomes. Three of these strains were isolated from clinical specimens of infected patients, including two MDR representatives of CC1 and CC2 and the sporadic isolate ATCC 17978. The fourth strain, SDF, has a non-human origin, although it has been suggested that its isolation from a body louse could result from the ingestion of contaminated blood from a bacteremic patient. While no significant differences in virulence were observed among the clinical strains, irrespective of their resistance phenotype or their classification as epidemic (AYE and ACICU) or sporadic (ATCC 17978), the body louse strain SDF was practically avirulent in this model of infection, suggesting that this strain could be used as a pathogenicity-defective benchmark in the search for novel *A. baumannii* virulence determinants.

In order to tentatively link the reduced virulence of SDF to the lack of specific virulence trait(s), the presence and expression of several virulence factors, namely hemolysins, phospholipase C, exoproteases and iron acquisition systems, were investigated by a combined approach of genomic and phenotypic analysis. In addition, several multifactorial phenotypes, such as growth capability, motility, biofilm formation and resistance to serum, iron deficiency and desiccation, were analyzed, as all these phenotypes are known to contribute to the pathogenicity and ecological fitness of other opportunistic bacterial pathogens.

On the whole, the *A. baumannii* clinical isolates displayed the ability to express a number of virulence determinants, including exoproducts with hemolytic, phospholipase and protease activities, and siderophore-based iron uptake mechanisms. This is in full agreement with the presence in their genomes of specific genes for the synthesis of such potential virulence factors [25], [27], [28], [29]. Notably, the hemolytic activity of *A. baumannii* strains is much more evident in liquid assay than on agar plates, and can be detected using horse, but not sheep, erythrocytes (Fig. 3). This intriguing result could explain why *A. baumannii* has historically been regarded as a non-hemolytic bacterium [43].

Differences were observed in the production of specific virulence factors between the non-human isolate SDF and the clinical strains, with the most noteworthy being the inability of SDF to use siderophores as an iron-uptake strategy, which affects its ability to grow under iron-depleted conditions (Fig. 2). On the other hand, SDF showed hemolytic, phospholipase and protease activities comparable to the average values obtained for the clinical strains (Figs. 3 and 4), suggesting that the reduced virulence of SDF in the *G. mellonella* model of infection cannot be ascribed to any of these factors. It should be pointed out that SDF showed limited metabolic capabilities, as inferred by its lower growth kinetics and biomass yields in the majority of culture media tested, as compared to the clinical *A. baumannii* strains (Fig. 1; data not shown). The genome of SDF is much smaller than that of the other *A. baumannii* genomes sequenced to date [5], presumably as a result of extensive insertion sequence-mediated deletion events [27]. Given that strain SDF was isolated from a human body louse, its adaptation to such a restricted host might provide an explanation for the reductive evolution of its genome and, consequently, for its metabolic restrictions, as documented for other pathogens restricted to a narrow ecological niche [reviewed in ref. 46]. Therefore, the virulence defect of SDF in *G. mellonella* can more likely be ascribed to a reduced ability to grow and persist in this model host than to impaired production of any virulence factor among those analyzed in the present study.

Some interesting differences were observed among the strains with respect to the multifactorial phenotypes related to pathogenesis, such as biofilm formation, motility and resistance to desiccation. The non-human isolate SDF was strongly impaired in its ability to survive on dry surfaces compared to the clinical isolates (Fig. 7B), suggesting that the ability to persist under dry conditions may represent a factor accounting for *A. baumannii* survival in the hospital setting and, ultimately, for its ability to cause outbreaks of infection.

A remarkable difference was observed between the representative CC1 and CC2 strains with regard to surface motility. While AYE displayed both twitching and swarming motilities, ACICU was apparently non-motile (Fig. 6), despite carrying the genetic potential to encode functional type IV pili (Table S2). Additional studies are required to assess whether the lack of motility is a specific feature of strain ACICU or a common trait of CC2 members.

Finally, it is worth noting that strain SDF is endowed with a surprisingly high capability to adhere to abiotic surfaces and to form biofilm. Very recently, de Breij and coworkers showed that no evident correlation exists in *A. baumannii* strains between biofilm formation capability and clinical impact [44]. The results obtained with SDF are in agreement with this conclusion. Intriguingly, the genome of SDF lacks most of the factors which have been implicated in *A. baumannii* biofilm formation, namely Csu pili, the quorum sensing signal synthase AbaI and exopolysaccharide PNAG synthesis enzymes (http://www.genome.jp/kegg/), suggesting either that these systems are not essential for biofilm development or that strain SDF possesses alternative compensatory function(s). However, a search for gene clusters exclusive to the genome of SDF did not reveal any obvious candidate(s), but a number of genomic regions of prophage origin with unpredictable function were identified. Whether these regions provide SDF with additional functions related to biofilm formation remains to be assessed.

In summary, the present work confirms the multifactorial nature of *A. baumannii* virulence, with no unique virulence factor being identified that individually accounts for the pathogenic success of this bacterium. Moreover, the results indicate that metabolic capabilities and resistance to environmental stresses might be more important for *A. baumannii* pathogenicity than the production of specific virulence factors. In support of this, it is noteworthy that genes coding for the only factors demonstrated to affect *A. baumannii* pathogenicity *in vivo* (OmpA, phospholipase D and penicillin-binding protein 7/8) are present in all annotated *A. baumannii* genomes, including the avirulent strain SDF, as well as in the genome of the related soil bacterium *Acinetobacter baylyi* (http://www.genome.jp/kegg/). The present investigation strengthens the view that the epidemic potential of *A. baumannii* is more likely related to the MDR phenotype than to the production of specific virulence factor(s) [1], [5], as shown here by the overall similarity of the virulence-related phenotype between epidemic MDR strains AYE and ACICU and the sporadic isolate ATCC 17978.

A comparative-genomics analysis recently demonstrated that the SDF genome is highly divergent from the genomes of clinical *A. baumannii* strains [5]. This work shows that such diversity reflects upon the atypical phenotype of SDF with regard to growth rate, iron uptake, biofilm formation and resistance to stress, compared with the clinical strains. Furthermore, the attenuated virulence in the insect model of infection argues against any potential role of SDF as a human pathogen. Further studies are mandatory to understand the actual distribution of such aberrant *A. baumannii* strains in reservoirs other than the hospital setting, which could possibly help to reconstruct the evolution of *A. baumannii* toward human pathogenicity.

## Supporting Information

Table S1 *A. baumannii* genomic ORFs predicted to encode hemolysin-, phospholipase- and exoprotease-related proteins.
(DOC)

Table S2 *A. baumannii* genomic ORFs predicted to encode proteins related to type IV pilus biogenesis and functioning.
(DOC)

Figure S1 Kaplan-Meier survival plots of *G. mellonella* larvae infected with the different *A. baumannii* strains. Time-kill results from a representative experiment are shown, which were obtained by inoculating 10^4^ (A), 10^5^ (B), 10^6^ (C) or 10^7^ (D) bacterial cells per larva. Strains are: AYE (dashed red lines), ACICU (straight green lines), ATCC 17978 (dashed blue lines) and SDF (straight black lines). Statistically significant differences (*P*<0.05 calculated by the log-rank test option of GraphPad) were only observed between SDF and the three clinical strains, but not between clinical strains.
(PPT)

Figure S2 Swarming-like motility on the air-agarose interface of ATCC 17978 on TSBD plates (A) and AYE on CAA plates (B) after 24 h of growth at 37°C. The plates are representative of three independent experiments giving similar results.
(PPT)
