# Supplementary material for: Deciphering the Multifactorial Nature of Acinetobacter baumannii Pathogenicity
Source: PLoS One. 2011 Aug 1;6(8):e22674. doi: 10.1371/journal.pone.0022674 (PMC3148234; doi:10.1371/journal.pone.0022674)
Supplement: Table S1 — A. baumannii genomic ORFs predicted to encode hemolysin-, phospholipase- and exoprotease-related proteins. (DOC) [file pone.0022674.s001.doc]

**Table S1.** *A. baumannii* genomic ORFs predicted to encode hemolysin-, phospholipase- and exoprotease-related proteins.

| **Predicted function(s)^a^** | **Strain^b^** | | | |
| --- | --- | --- | --- | --- |
|  | **AYE** | **ACICU** | **ATCC 17978** | **SDF** |
| Putative hemagglutinin/hemolysin-related protein (no signal peptide, OM) | - | - | - | 3544 |
| Putative hemolysin (no signal peptide, unknown) | 2926 | 0840 | 0889 | 2547 |
| Putative hemolysin-related protein (no signal peptide, IM) | 2786 | 0966 | 1005 | 2385 |
| Hemolysin-type calcium-binding region (no signal peptide, extracellular) | - | - | 1073 | - |
| Putative hemolysin (signal peptide, extracytoplasmic) | 2389 | 1322 | 1321 | - |
| Hemagglutinin/hemolysin-related protein (no signal peptide, OM) | - | 1911 | - | - |
| Putative hemolysin-type calcium-binding region (no signal peptide, extracellular) | - | - | 2413 | - |
| Putative calcium binding hemolysin protein (no signal peptide, extracellular) | - | - | 2414 | - |
| Hemolysin-type calcium-binding protein with a RTX N-terminal domain (no signal peptide, OM or extracellular) | - | 2938 | 2696 | - |
| Putative hemolysin III (no signal peptide, IM) | 0623 | 3107 | 2859 | 0585 |
|  |  |  |  |  |
| Phospolipase C (signal peptide, extracellular) | 3825 | 0064 | 0043 | 0054 |
| Phospolipase C (signal peptide, extracellular) | 1520 | 2247 | 2055 | 2207 |
|  |  |  |  |  |
| Serine protease (signal peptide, extracellular) | 1602 | 2073 | 1954 | - |
| Putative aminopeptidase N (signal peptide, extracellular) | 1122 | 2554 | 2356 | 1167 |
| Putative zinc protease (signal peptide, OM) | 990 | 2679 | 2470 | 1071 |
| Serine protease (signal peptide, OM) | 359 | 3327 | 3126 | 363 |

^a^ The presence of a signal peptide for protein export into the periplasm and the subcellular localization of each protein have been predicted by the PSORTb program (<http://www.psort.org/>), and are shown in brackets. Abbreviations; OM, outer membrane; IM, inner membrane.

^b^ Number of genes refers to the annotation of each genome sequence (Kyoto Encyclopedia of Genes and Genomes; http://www.genome.jp/kegg/).
