# Supplementary material for: Deciphering the Multifactorial Nature of Acinetobacter baumannii Pathogenicity
Source: PLoS One. 2011 Aug 1;6(8):e22674. doi: 10.1371/journal.pone.0022674 (PMC3148234; doi:10.1371/journal.pone.0022674)
Supplement: Table S2 — A. baumannii genomic ORFs predicted to encode proteins related to type IV pilus biogenesis and functioning. (DOC) [file pone.0022674.s002.doc]

**Table S2.** *A. baumannii* genomic ORFs predicted to encode proteins related to type IV pilus biogenesis and functioning.

| **Gene** | **Predicted function** | **Strain^a^** | | | |
| --- | --- | --- | --- | --- | --- |
|  |  | **AYE** | **ACICU** | **ATCC 17978** | **SDF** |
|  |  |  |  |  |  |
| *pilR* | Type 4 fimbriae expression regulatory protein | 3535 | 0257 | 0234 | - |
| *pilS* | Sensor protein | 3536 | 0258 | 0235 | - |
|  |  |  |  |  |  |
| *pilD* | Type 4 prepilin-like proteins leader peptide processing enzyme | 3446 | 0343 | 0327 | 3194 |
| *pilC* | Type 4 fimbrial assembly protein | 3445 | 0344 | 0328 | - |
| *pilB* | Type 4 fimbrial biogenesis protein | 3444 | 0345 | 0329 | - |
|  |  |  |  |  |  |
| *pilF* | Type 4 fimbrial biogenesis protein | 3265 | 0509 | 0500 | - |
|  |  |  |  |  |  |
| *pilU* | Twitching motility protein | 2919 | 0847 | 0896 | - |
| *pilT* | Twitching motility protein | 2918 | 0848 | 0897 | - |
|  |  |  |  |  |  |
| *pilZ* | Type 4 fimbrial biogenesis protein | 2074 | 1606 | 1559 | 1726 |
|  |  |  |  |  |  |
| *pilJ* | Type IV pilus biogenesis protein | 0670 | 3060 | 2812 | 0641 |
| *pilI* | Twitching motility protein | 0669 | 3061 | 2813 | 0640 |
| *pilH* | Twitching motility two-component system response regulator | 0668 | 3062 | 2814 | 0639 |
| *pilG* | Twitching motility two-component system response regulator | 0667 | 3063 | 2815 | 0638 |
|  |  |  |  |  |  |
| *fimT* | Type IV pilus assembly protein | 0639 | 3090 | 2841 | 0604 |
|  |  |  |  |  |  |
| *pilE* | Type IV pilus assembly protein | 0320 | 3364 | 3165 | 0325 |
| *pilE* | Pilin like competence factor | 0319 | 3365 | 3166 | - |
| *pilY1* | Type IV pilus assembly protein | 0318 | 3366 | 3167 | - |
| *pilX* | Putative type IV fimbrial biogenesis protein | 0317 | 3367 | - | - |
| *pilW* | Type IV pilus assembly protein | 0316 | 3368 | 3168 | - |
| *pilV* | Type IV fimbrial biogenesis protein | 0315 | 3369 | - | - |
| *pilT* | Putative type IV fimbrial biogenesis protein | 0314 | 3370 | - | - |
|  |  |  |  |  |  |
| *pilA* | Major pilin | 0304 | 3380 | 3177 | - |
|  |  |  |  |  |  |
| *pilQ* | Type IV pilus assembly protein | 0294 | 3390 | 3191 | - |
| *pilp* | Type IV pilus assembly protein | 0293 | 3391 | 3192 | 0297 |
| *pilO* | Type IV pilus assembly protein | 0292 | 3392 | 3193 | 0296 |
| *pilN* | Type IV pilus assembly protein | 0291 | 3393 | 3194 | - |
| *pilM* | Type IV pilus assembly protein | 0290 | 3394 | 3195 | 0293 |
|  |  |  |  |  |  |

^a^ Number of genes refers to the annotation of each genome sequence (Kyoto Encyclopedia of Genes and Genomes; http://www.genome.jp/kegg/).
