# Supplementary material for: Deciphering the Multifactorial Nature of Acinetobacter baumannii Pathogenicity
Source: PLoS One. 2011 Aug 1;6(8):e22674. doi: 10.1371/journal.pone.0022674 (PMC3148234; doi:10.1371/journal.pone.0022674)
Supplement: Figure S1 — Kaplan-Meier survival plots of G. mellonella larvae infected with the different A. baumannii strains. Time-kill results from a representative experiment are shown, which were obtained by inoculating 104 (A), 105 (B), 106 (C) or 107 (D) bacterial cells per larva. Strains are: AYE (dashed red lines), ACICU (straight green lines), ATCC 17978 (dashed blue lines) and SDF (straight black lines). Statistically significant differences (P<0.05 calculated by the log-rank test option of GraphPad) were only observed between SDF and the three clinical strains, but not between clinical strains. (PPT) [file pone.0022674.s003.ppt]

## Slide 1
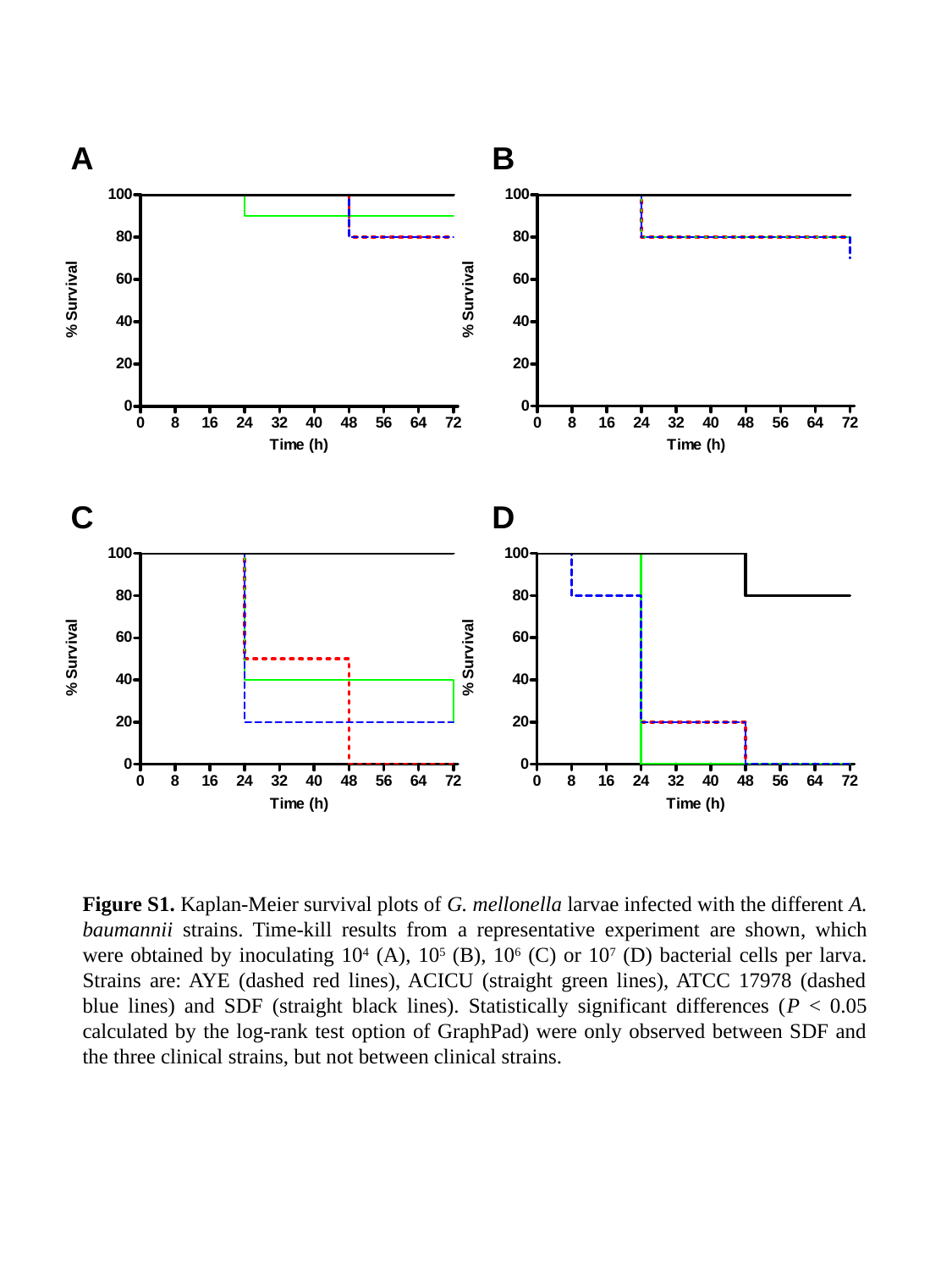

A
B
C
D
Figure S1. Kaplan-Meier survival plots of G. mellonella larvae infected with the different A. baumannii strains. Time-kill results from a representative experiment are shown, which were obtained by inoculating 104 (A), 105 (B), 106 (C) or 107 (D) bacterial cells per larva. Strains are: AYE (dashed red lines), ACICU (straight green lines), ATCC 17978 (dashed blue lines) and SDF (straight black lines). Statistically significant differences (P < 0.05 calculated by the log-rank test option of GraphPad) were only observed between SDF and the three clinical strains, but not between clinical strains.
