# Supplementary material for: Deciphering the Multifactorial Nature of Acinetobacter baumannii Pathogenicity
Source: PLoS One. 2011 Aug 1;6(8):e22674. doi: 10.1371/journal.pone.0022674 (PMC3148234; doi:10.1371/journal.pone.0022674)
Supplement: Figure S2 — Swarming-like motility on the air-agarose interface of ATCC 17978 on TSBD plates (A) and AYE on CAA plates (B) after 24 h of growth at 37°C. The plates are representative of three independent experiments giving similar results. (PPT) [file pone.0022674.s004.ppt]

## Slide 1
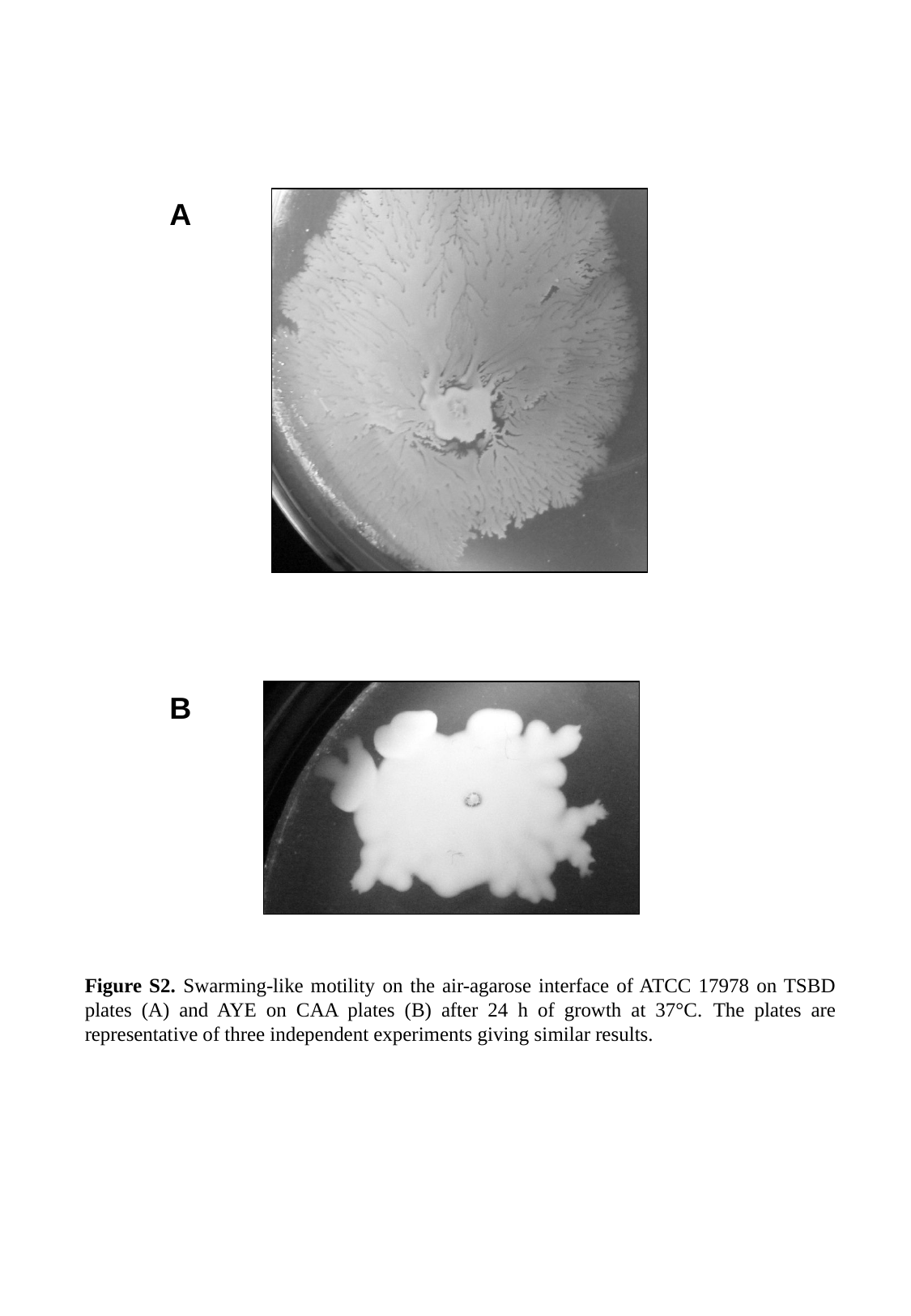

A
B
Figure S2. Swarming-like motility on the air-agarose interface of ATCC 17978 on TSBD plates (A) and AYE on CAA plates (B) after 24 h of growth at 37°C. The plates are representative of three independent experiments giving similar results.
